# Editorial

**DOI:** 10.1120/jacmp.v3i2.2574

**Published:** 2002-03-01

The American College of Medical Physics (ACMP) and the Journal of Applied Clinical Medical Physics (JACMP) are very pleased to announce that with this issue the JACMP is now an official journal of the International Organization for Medical Physics (IOMP). As an all‐electronic journal and with the current non‐subscription policy the journal is available and accessible to medical physicists world‐wide. From the beginning of publication over two years ago, it was clear that the journal was being accessed in many different parts of the world. About 20% of the manuscripts submitted are from outside the United States, and to date papers from over half‐a‐dozen countries have been published. It has been the editorial policy of the journal to encourage this participation, and this agreement to become an official journal of the IOMP will further strengthen our ties with the international community of medical physicists.

This concept was initiated by the Board of Editors a year ago and was presented to the Board of Chancellors of the ACMP at the annual meeting in June of 2001. With their concurrence the Editor‐in‐Chief proceeded with discussions with Gino Fallone, Chairman of the IOMP Publications Committee. This resulted in the invitation by Oskar Chomicki, the President of the IOMP, for the JACMP to become an official journal of the IOMP. The ACMP and the JACMP wish to express their thanks to the IOMP and its officials for their constructive discussions that made this possible.

Under the terms of the agreement the Editor‐in‐Chief of the JACMP will be an ex officio member of the IOMP Publications Committee and a representative of the IOMP will be invited to attend the Editorial Board meetings of the JACMP.

The ACMP and the JACMP look forward to many years of fruitful cooperation with the IOMP.

Peter R. Almond, Ph.D.

Editor‐in‐Chief

